# C2 Laminar screw—screw assembly for the cranio-cervical junction

**DOI:** 10.3389/fsurg.2026.1726576

**Published:** 2026-06-15

**Authors:** Wei Song, Haihong Zhang, Jing Wang

**Affiliations:** 1 The Second Hospital & Clinical Medical School, Lanzhou University, Lanzhou, Gansu, China; 2 Department of Orthopedics, Orthopedics Key Laboratory of Gansu Province, Lanzhou, Gansu, China; 3 Department of Orthopedics, Minxian County People’s Hospital, Dingxi, China

**Keywords:** atlantoaxial dislocation, C2 laminar screws, craniocervical junction, high-riding vertebral artery, screw—screw assembly

## Abstract

Atlantoaxial dislocation is a common disorder of the upper cervical spine that often requires surgical treatment. The primary treatment methods include transoral release and fixation, as well as posterior screw-rod fixation. When patients have anatomical variations in the atlas and axis, the difficulty of screw placement is significantly increased. Therefore, we have adopted a new laminar screw-screw fixation technique to replace the traditional C2 screw fixation. This method has been used to treat atlantoaxial dislocation.

## 1 Introduction

When C1 pedicle screws are utilized for fixation, and anatomical variations in C2 prevent the use of traditional C2 screws, a C2 laminar screw is then employed. Since the C1 pedicle screws and C2 laminar screw are not aligned in the same plane, an additional pedicle screw is inserted to connect with the C2 laminar screw. This arrangement ensures that the heads of the pedicle screws are aligned in the same plane, thereby facilitating the final connection of the rod.

## 2 Background

The concept of craniocervical deformities and atlanto-occipital joint dislocations was first introduced by Blackwood in 1908. These conditions are primarily caused by factors such as trauma, rheumatoid arthritis, infections, tumors, congenital abnormalities, and degenerative diseases (1). Surgical treatment for upper cervical spine conditions typically involves anterior or posterior approaches. Among these, posterior fixation and fusion of the atlantoaxial joint is a key procedure. Over time, surgical techniques have evolved from the initial cable fixation to the widely used posterior screw-rod systems. Common methods include C1 lateral mass screws combined with C2 pedicle screws, or occipital plates with C1 lateral mass screws and C2 pedicle screws. These foundational techniques have further developed to address complex cases of atlantoaxial instability. The anatomy of the upper cervical spine, characterized by significant variability in neural and vascular structures, presents considerable risks during pedicle screw placement. For instance, a high-riding vertebral artery increases the likelihood of arterial injury during screw insertion (2–4). An alternative approach involves extending the fusion to the lower cervical spine. However, this method significantly reduces the patient's cervical spine mobility. In 2004, Wright first introduced the technique of C2 laminar screw fixation (5), This technique effectively minimizes the risk of vertebral artery injury and allows direct visualization of screw placement during surgery. It serves as an alternative approach for patients with vertebral artery anomalies (6–8). Some surgeries involve extending fixation beyond the occipito-cervical junction to lower segments. When laminar screws are placed in the C2 vertebra, misalignment of screw heads across segments can complicate rod connection. This requires either bending the rod into a precise curve, increasing surgical time and complexity, or using specialized connectors (9). However, these specialized connectors are often difficult to obtain, and they do not allow for directional adjustments of the rod ends. To address these challenges, we propose using a screw-to-screw connection technique.

## 3 Surgical technique

### 3.1 Occipital plate placement

Position the occipital plate below the external occipital protuberance along the midline.

Prepare the bone by drilling and removing the cortical layer at the locations of the three screw holes.

Insert three occipital screws to secure the plate.

### 3.2 Atlas lateral mass screws

The procedure for placing these screws is not detailed in the provided text. It typically involves identifying the lateral mass of C1 and drilling into it at a specific angle to avoid neurovascular structures.

### 3.3 Axis pedicle screws

Identify the external border of the spinal canal and the inner edge of the pedicle.

Start the entry point 2 mm below and outward from this location.

Using a drill and a curette, follow the trajectory of the pedicle at an inward angle of about 20–25° and an upward tilt of 25–30°.

Use depth gauges to create the screw path gradually.

Tap the path and then insert polyaxial screws.

### 3.4 Axis lamina screws

For one side, the entry point is where a line extended from the lamina to the opposite spinous process intersects 4 mm below the lamina edge. On the other side, the intersection point is 7 mm below the lamina edge.

After opening the cortical bone with a burr, use a curette directed towards the opposite articular process and parallel to the lamina direction to create the screw path.

Insert two polyaxial screws in a crossing pattern.

### 3.5 C3 Lateral mass screws

The entry point is 1 mm inward and downward from the midpoint of the lateral mass.

After opening the cortical bone, use a curette along with a depth gauge to direct the drill toward the outer upper quadrant to create the screw path.

Insert a polyaxial screw.

### 3.6 Domino connection using polyaxial screws

Use polyaxial screws to connect the screw bodies with the lamina screws.

Adjust the length of the connecting screws to ensure that the thicker parts connect with the C2 lamina screws, facilitating robust and secure assembly.

This approach optimizes the stability and alignment of the construct, while minimizing risks associated with traditional rod placement and bending.

**Figure 1:**
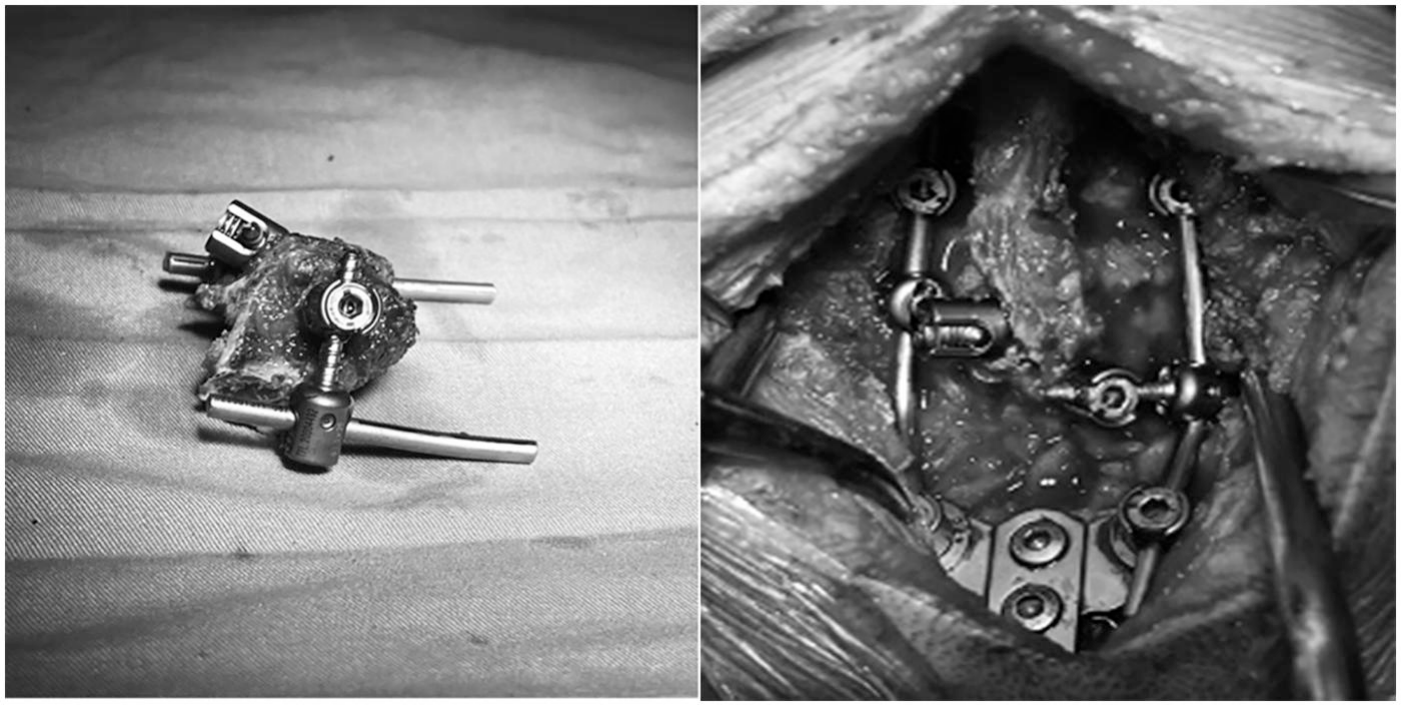
Intraoperative demonstration of the C2 laminar screw–screw assembly technique. Crossed C2 laminar screws were inserted through the lamina, and an additional polyaxial screw was used as a screw-to-screw domino connector to align the screw heads and facilitate rod connection at the cranio-cervical junction.

**Figure 2:**
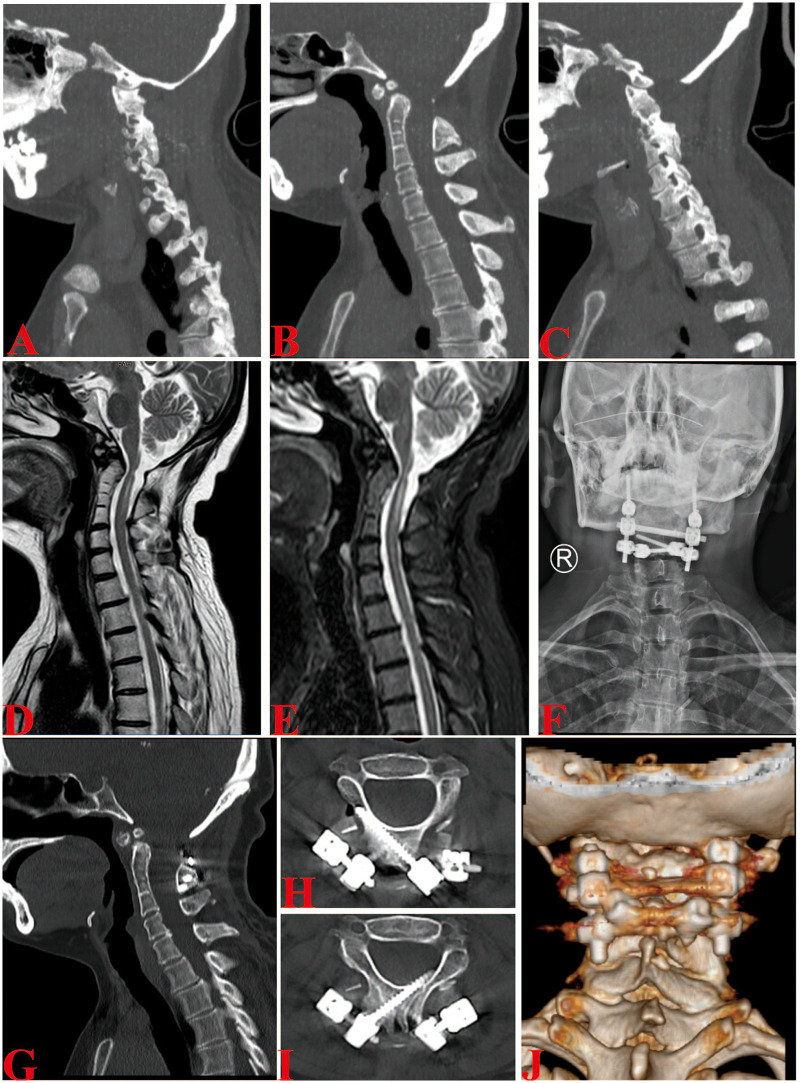
A 45-year-old female: **(A–C)** preoperative CT showed odontoid process dissociation and atlantoaxial dislocation; **(D,E)** the spinal cord was obviously compressed and thinned on MRI before operation; **(F)** x-ray at 3 days after operation: **(G–J)** it was 2 years after operation, and CT showed no vertebral lamina screws or loosening between screws.

**Figure 3:**
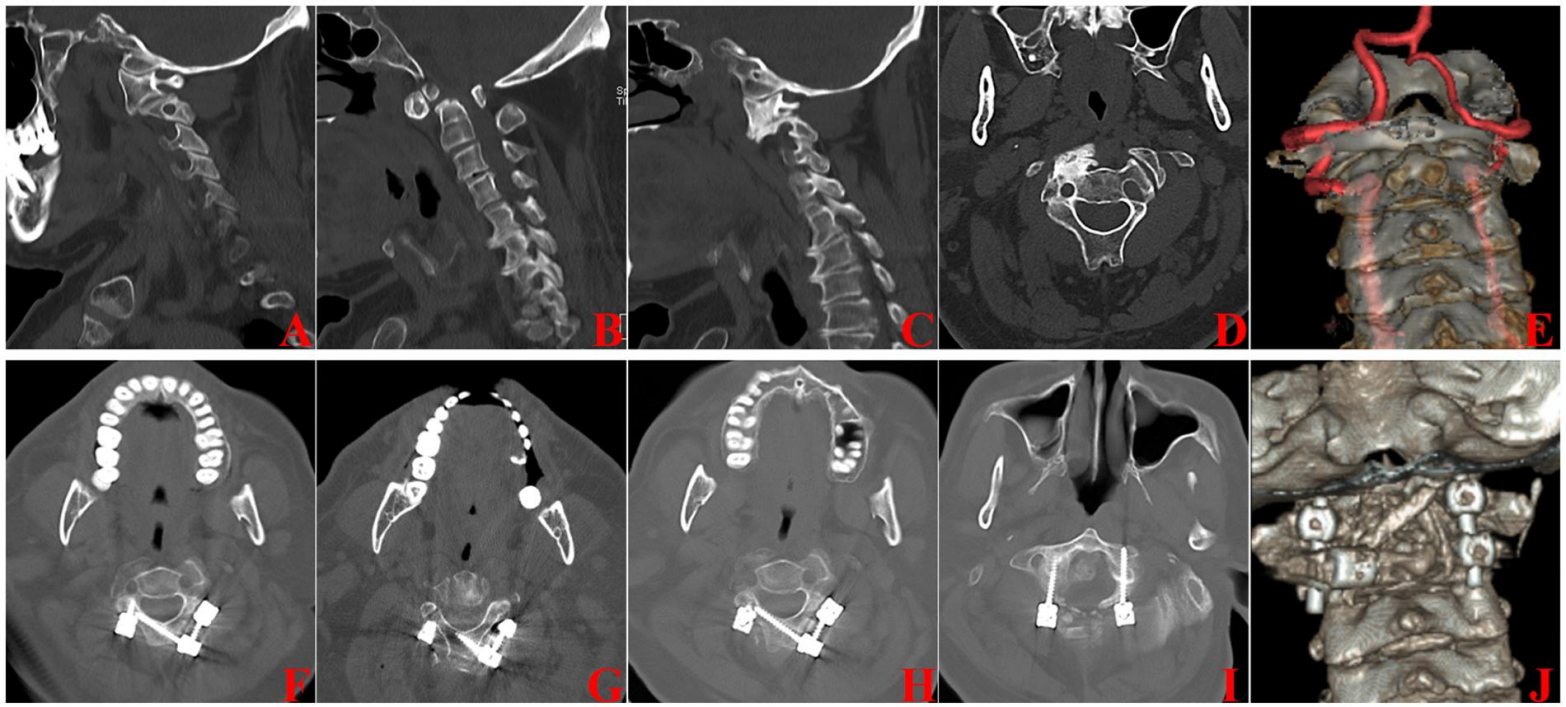
A 48-year-old male: **(A–E)** CT and vertebral arteriography showed atlantoaxial dislocation, free odontoid process and high span vertebral artery; **(F–J)** CT performed 2 years after surgery revealed well-positioned C2 laminar screws with intact screw-to-screw connections.

**Figure 4:**
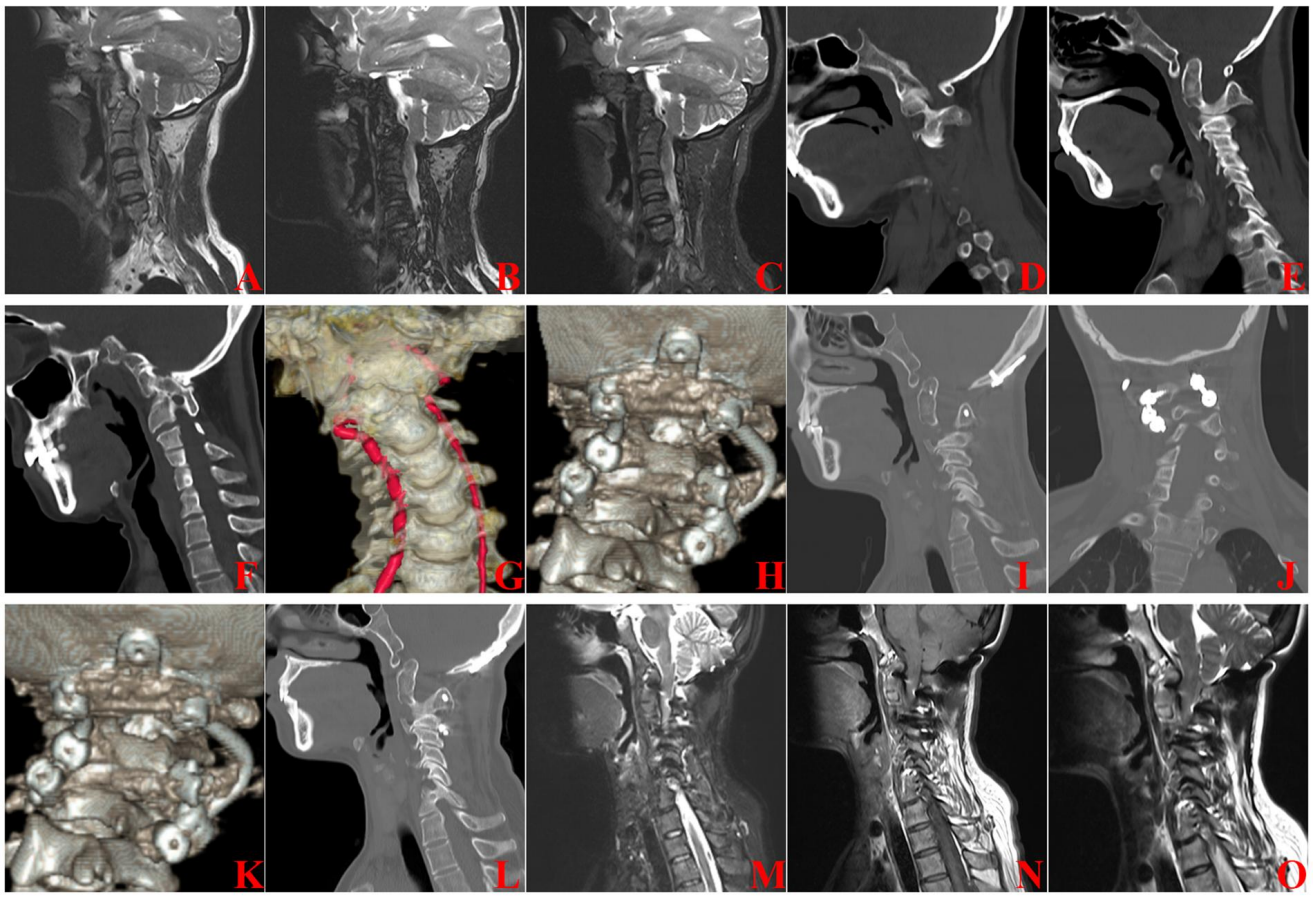
A 44-year-old female: **(A–C)** preoperative sagittal MRI revealed atlantoaxial dislocation with marked spinal cord compression; **(C–G)** preoperative CT demonstrated basilar invagination, occipito-cervical fusion, atlantoaxial dislocation, C2–3 vertebral block, and a high-riding vertebral artery; **(H–J)** CT at 1 year post-op confirmed satisfactory reduction of the atlantoaxial dislocation; **(K–O)** CT and MRI at 1.5 years both showed persistent decompression of the spinal cord.

Adjusting the angle and length of the domino screws, the next step is to connect the rods, as shown in Figure 1. Representative cases demonstrating the application of this technique are shown in Figures 2–4.

## 4 Discussion

In 1927, Foerster was the first to perform surgery in the cranio-cervical junction area. Following his pioneering work, some surgeons attempted to achieve stabilization by using bone grafting and securing the atlantoaxial vertebrae with wire binding. However, these methods did not provide satisfactory stability (10). Since the 1990s, posterior rod-wire fixation combined with posterior bone grafting techniques has been applied to the cranio-cervical junction area. This approach has significantly improved stability and fusion rates compared to earlier fixation methods (11). Subsequent research has shown that this technique, while initially promising, offers less stability compared to posterior screw-rod fixation. Additionally, it poses a higher risk of complications, such as spinal cord and nerve injuries (12–14). In 1991, Grob reported on the internal fixation technique combining an occipital plate with atlantoaxial lateral mass screws, known as the screw-plate system. This method provided a more robust and reliable means of achieving stabilization at the cranio-cervical junction (15). This technique, introduced by Grob, offers improved stability and eliminates the need for post-operative external bracing, which was a significant advancement over previous methods. However, it does not allow for distraction or realignment, which can result in suboptimal anatomical restoration of the cranio-cervical junction. In 1999, Abumi introduced a further advancement with the use of an occipital plate and axis pedicle screws in a screw-rod system for cranio-cervical internal fixation. This newer method provided additional stability and the capability to better align the cranio-cervical anatomy during surgery (16). The screw-rod fixation technique allows for distraction or compression, facilitating better realignment of the bony structures in the cranio-cervical area. Initially, the fixation screws of the occipital plate were placed on either side, but given the structural characteristics of the occiput, this only provided limited fixation strength. Technological advancements led to the placement of fixation screws along the midline of the occiput, where the bone is thicker and can provide greater resistance to pull-out forces and more robust fixation strength. This adjustment has enhanced the postoperative fusion rates of the cranio-cervical junction. Modern internal fixation systems commonly use polyaxial screws with diameters of 3.5 or 4 mm, matched with rods that can be easily bent to accommodate varying screw placements and the natural curvature of the cranio-cervical area. These systems offer sufficient stability, reduce the need for external bracing, significantly improve fusion rates, and possess the advantage of stabilizing only the unstable segments without affecting the overall mobility of the spine (17–19). The classic Magerl technique, which involves internal fixation through atlantoaxial lateral mass screws, is employed in occipitocervical fusion. This method is recognized for providing superior stability and safety. However, there is a risk of vertebral artery injury with this technique, especially in patients with vertebral artery anomalies, when placing both the axis pedicle screws and the lateral mass screws. Careful preoperative imaging and planning are essential to minimize this risk and ensure the safety of the procedure (20).

In 2004, Wright pioneered the use of laminar screw fixation, applying this technique to treat 10 patients. Follow-up assessments showed favorable outcomes, with no severe complications reported. This method demonstrated its effectiveness and safety in clinical practice (5). The primary advantage of this fixation method is that the position of the screws is away from the vertebral artery, significantly reducing the risk of arterial injury. Moreover, the entire screw placement process can be performed under direct visualization, which simplifies the procedure and enhances safety. As long as the axis lamina is normally developed, screws can be placed, making it an effective alternative to pedicle screw techniques, especially in cases where there is abnormal development of the axis pedicle or atypical positioning of the vertebral artery foramen. Numerous biomechanical studies have focused on the C2 laminar screw fixation technique. In one such study by Gorek, biomechanical experiments were conducted on fresh human cadaveric specimens. These studies involved creating models of odontoid process fractures and then stabilizing them with C1-C2 internal fixation. By comparing the stability provided by bilateral pedicle screws, unilateral pedicle screw combined with a unilateral laminar screw, and bilateral laminar screws, it was concluded that bilateral laminar screws can offer stability comparable to that of pedicle screws. This finding underscores the viability of laminar screws as a robust alternative for cervical spine stabilization, especially in scenarios where pedicle screw placement might be risky or impractical (21).

The entry points for laminar screws are located at the base of the spinous processes, whereas pedicle screws and occipital plates are positioned more laterally, making the connection of the rods more challenging. Therefore, the rods require bending in both the sagittal and coronal planes to accommodate the cervical curvature and the distance between the screws, ensuring better alignment and stabilization. Using connecting rods can simplify this process by eliminating the need for secondary bending of the rods; only bending in the sagittal plane is necessary. This not only reduces the complexity of rod placement but also decreases the risk of rod breakage during post-operative periods, enhancing the overall durability and reliability of the internal fixation.

In the current environment of centralized procurement, acquiring specific connectors that are also adaptable in direction can be challenging. These challenges pose new hurdles for clinical practice. To address these issues, a screw-to-screw connection method has been adopted, which offers several advantages: (1) Ease of Availability: This method does not require specialized connectors. Instead, it uses readily available universal screws for connections. (2) Simplified Rod Placement: The use of universal screws significantly simplifies the rod placement process, effectively reducing surgical time by eliminating the need for precise alignment of specialized connectors. This approach not only streamlines the procedure but also enhances the flexibility and efficiency of spinal surgeries, adapting well to the limitations posed by procurement constraints. Several C2 fixation techniques have been applied in cranio-cervical junction surgery, each with specific advantages and limitations. To further clarify the clinical applicability of the C2 laminar screw–screw assembly technique, the advantages and disadvantages of different C2 fixation methods are summarized in Table 1. This table summarizes the main advantages and disadvantages of commonly used C2 fixation techniques, with particular emphasis on biomechanical stability, surgical difficulty, applicability, and the risk of vertebral artery or neurovascular injury.

**Table 1:** Compare the advantages and disadvantages of different C2 nail placement methods.

C2 Nailing method	Advantage	Disadvantage
C2 Laminar screw—screw Assembly	Reduces the risk of vertebral artery injury	Insufficient observation of spinal cord injury risk
		Slightly weaker fixation streng
	Relatively low technical requirements	
	Good biomechanical stability	
	Wide range of applicability	
C2 Pedicle screw	Strong biomechanical stability	High risk of vertebral artery injury
		High technical requirements
C2 Isthmus screw	Good biomechanical stability	Slightly weaker fixation strength
	Strong adaptability	limited range of applicability
	Relatively simple operation	
C2 Spinous process screw	Relatively simple operation	Relatively weaker fixation strength
	minimal damage to surrounding structures	additional fixation methods
C2–C3 Transfaceta	Strong biomechanical stability	High technical requirements
	Provides multiplanar fixation	Potential damage to surrounding neurovascular structures

This table summarizes the main advantages and disadvantages of commonly used C2 fixation techniques, with particular emphasis on biomechanical stability, surgical difficulty, applicability, and the risk of vertebral artery or neurovascular injury.

## 5 Technical limitations

When osteoporosis or osteoarthritis is present, there is a risk of entering the spinal canal when using a milling drill to make grooves and subsequently using an open-path vertebra. Moreover, when the head rotates, the stress on the lamina from the screws increases, which in turn raises the risk of screw protrusion into the spinal canal. The length of screw implantation should not be excessive, as overly long screws will increase the risk of vertebral artery injury. When posterior bone grafting is required, the cortical bone of the C2 lamina should not be removed too much, which requires a certain level of experience from the surgeon; otherwise, the holding power of the laminar screw will be significantly reduced. Additionally, the stability of the connection between screws, due to the reasons of screw threads, needs further biomechanical experimental verification. In special cases, it needs to be determined whether the screw threads need to be ground.

## 6 Conclusion

In upper cervical spine surgeries, especially for patients with C2 vertebral anomalies or difficulties in screw placement, the use of a screw-to-screw domino connection combined with C2 laminar cross screws offers biomechanical effects similar to those of laminar screws connected with rods and C2 pedicle screws. The screw-to-screw domino method facilitates easier adjustments of connection length and angle due to the use of universal screws, making rod connection more straightforward. Therefore, in scenarios where pedicle screws cannot be implanted or connecting rods are unavailable, the technique of C2 laminar screws with screw-to-screw domino connections can serve as an effective alternative. This method provides sufficient biomechanical stability while offering greater flexibility and adaptability in surgical application, addressing the challenges of specific anatomical variations or procurement limitations.

## Data Availability

The raw data supporting the conclusions of this article will be made available by the authors, without undue reservation.
